# Physical, Perceptual, Socio-Relational, and Affective Skills of Five-Year-Old Children Born Preterm and Full-Term According to Their Body Mass Index

**DOI:** 10.3390/ijerph18073769

**Published:** 2021-04-04

**Authors:** Pedro Gil-Madrona, Sonia J. Romero-Martínez, Carmen C. Roz-Faraco

**Affiliations:** 1 Faculty of Education, Albacete, University of Castilla La Mancha, 13001 Ciudad Real, Spain; 2 Faculty of Psychology, National University of Distance Education, 28015 Madrid, Spain; sjromero@psi.uned.es; 3 Faculty of Education, University Antonio of Nebrij, 28015 Madrid, Spain; croz@nebrija.es

**Keywords:** preterm children, physical-motor skills, perceptual-motor skills, socio-relational skills, body mass index

## Abstract

The main purpose of this study was to compare the psychomotor development of five-year-old children born preterm and full term. The comparison included physical-motor, perceptual-motor, and socio-relational and affective skills. As low weight is one of the variables that most influences the psychomotor development of premature infants, a secondary aim was to analyze these skills according to their current body mass index (BMI). A prospective simple ex-post facto study was conducted. The sample consisted of 672 five-year-old children enrolled in the third year of early childhood education in the province of Albacete, Spain; 35 of them was born prematurely. Children were evaluated by their teachers using the Checklist of Psychomotor Activities (CPA). The results show that children born preterm had a lower development of their physical-motor skills. In the perceptual-motor field, premature children showed lower scores in the variables related to their body image and body schema, motor dissociation, and visual-motor coordination, as well as in socio-relational and affective aspects. However, the development in laterality, dynamic coordination, motor execution, tonic-postural control, and balance were not affected. These differences were not affected by the current weight, given that the analysis of the BMI indicated no differences in preterm children. This study demonstrated the need to establish protocols oriented to the prevention of the difficulties detected in children with psychomotor high-risk and the needs to reinforce the educational programs in this area to improve the integral development of children born preterm.

## 1. Introduction

Preterm birth is defined as onset of labor prior to the completion of 37 weeks of gestation in a pregnancy beyond 20 weeks of gestation. The period of viability varies in different countries from 20 to 28 weeks. Developmental delays in premature infants have been widely documented; however, there are factors that may mediate or moderate the impact of prematurity on future development, and some of them are related to the environment, such as the family [1] or the school [2]. The present study was centered on the second group of factors mentioned above.

The assessment of psychomotor skills is necessary in preterm and full-term children to establish what the physical-motor, perceptual-motor, socio-relational, and affective skills are that are important to inform the design of educational activities in terms of contributing to preventing developmental difficulties more common in premature infants. In preterm children, the physical-motor aspect is one of the most important areas for their development at an early age due to its close relationship with the cognitive, emotional, and affective factors of children [3], being fundamental in preventive settings. Several authors agree that the term psychomotricity implies motor development, and this in turn implies physical changes and interaction with the context, resulting in the evolution of other processes such as cognitive and affective factors [4,5]. Very premature newborns have a particularly high risk of suffering a significant variety of neurodevelopmental disorders, including cerebral palsy (CP) and sensory, cognitive, and behavioral disabilities. Therefore doctors, teachers, and relatives must observe the child in all the evolutionary stages, monitoring their motor, socio-emotional, and cognitive development.

When children with a possible psychomotor retardation are observed, it is necessary to activate referral mechanisms to provide them with early intervention. When monitoring premature children, the importance of the observation of their skills in the first years of life (between zero and two) is stressed, despite of absence of motor deficiencies that may occur after this age [6,7,8]. It is evident that there is a strong relationship between motor deficiencies, if any, and subsequent cognitive dysfunction, which supports the hypothesis that these difficulties have a common origin [9]. For this reason, it is important to highlight the importance of detection and follow-up of motor and cognitive deficiencies in preterm children [10], with deficiencies being the cause of great vulnerability when they are not diagnosed. In recent studies, it has been determined that preterm children can maintain a disability-free survival, but the consequences that lead to socio-affective, motor, and cognitive problems have not been deeply studied [11].

Prematurity in children is identified as one of the causes of high biological risk detected by a multidisciplinary observation process [12]. It should be noted that more than 1 in 10 babies are born prematurely worldwide; this rate is increasing, with an incidence of more than 15 million children born prematurely in the world. In addition to these alarming data, more than 1 million children die each year due to complications during preterm birth, and others that survive live with disability, including learning, motor, visual, and hearing problems [13].

Observation and prevention of risk factors are key aspects in the identification of psycho-motor problems in premature children who may be disadvantaged compared to non-premature children [14]. The development of motor, perceptual, socio-relational, and affective skills is a very important aspect in childhood development, and it should be approached at an early age, being the basis for acquiring aspects that support the executive functions of infants [15].

These results highlight the importance of prevention and the establishment of referral, observation, and follow-up protocols in preterm children to different areas such as medicine, education, and psychology for the implementation of actions that favor children’s motor development [16]. Although many studies have assessed the motor development of premature children [17], only a few have been conducted that take into consideration an older school age group such as the age of five-years-old, when the initial deficiencies appear in the acquisition of cognitive and social skills.

On the other hand, previous research [15,16,17] has found that premature children exhibit lower weight compared with full-term children, as was the case in the present study. Lower weight has also been associated with a delay in the psychomotor development in some studies [13,14], and for this reason, the hypothesis of the present study is that the weight, in conjunction with the condition of premature birth, could have a conditional effect on the psychomotor development.

This study’s main objective was to compare the psychomotor development and the socio-emotional aspects of five-year-old children in order to check the aforementioned hypothesis, taking in consideration their characteristics of birth (preterm or full-term). A secondary objective was to analyze the psychomotor skills in both preterm and full-term children, taking into account their weight measured by current body mass index (BMI). This age was chosen because it is a crucial stage between early childhood education and primary education since it is when other psychomotor and socio-emotional skills are developed.

The results of the study could allow for the identification of physical-motor, perceptual-motor, and socio-emotional factors influenced by the characteristic of being children born preterm or full-term. These factors would allow for the design of preventive and corrective actions focused on the promotion of the holistic and harmonious development of premature children before they start the stage of elementary school.

## 2. Materials and Methods

### 2.1. Sample

**Figure 1:**
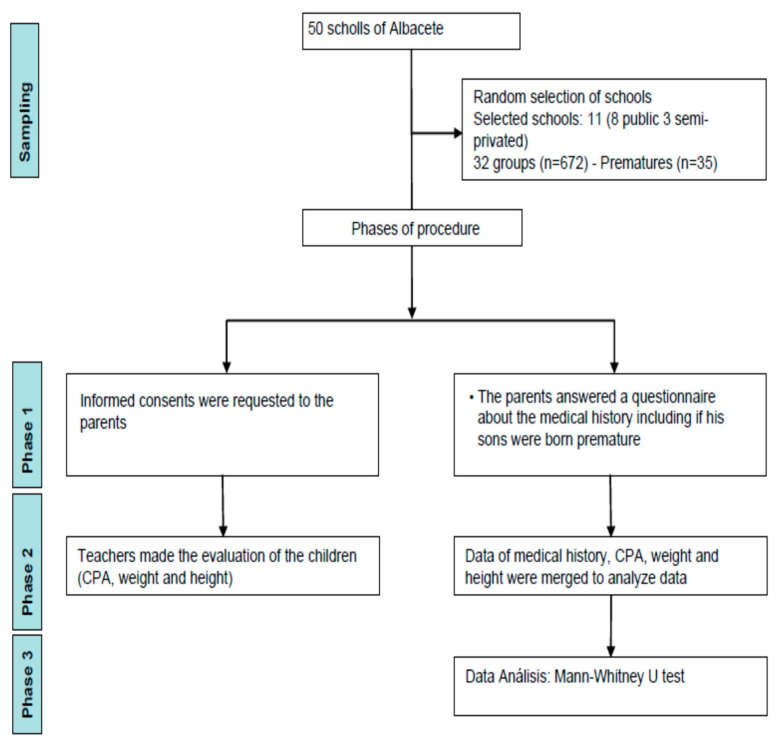
Phases of the procedure and sampling. Note: CPA = Checklist of Psychomotor Activities.

The sample was composed by 672 children aged 5 years old, enrolled in the third level of early childhood school. Data were gathered by teachers of 32 groups of students in 11 schools in the province of Albacete (Spain). The subsample of 11 participating schools were randomly selected from a population of 50 schools, located in the aforementioned province during the year 2018 according to the city council record [18] (see Figure 1). To gather the data, we requested the consent of the parents, guaranteed anonymity and confidentiality, and followed the ethical principles for medical research involving human subjects (Declaration of Helsinki) [19].

In total, 46.7% of the sample were girls and 53.3% were boys. We found that 5.2% of the total sample (*n* = 35) were born preterm in 2012; this information matches with the reports of Save the Children and the World Health Organization, stating that the rate in Spain was 7.14 out of 100 preterm births in the same year [20]. Prematurity definition for this study is considered in the above-mentioned reports (children born before week 37).

Children were classified according to BMI into 5 groups according to the conventional World Health Organization classification [21] for 5-year-old boys: severely underweight (<12.1 kg/m^2^), underweight (12.2–12.9 kg/m^2^), normal weight (13–16.6 kg/m^2^), overweight (16.7–19.9 kg/m^2^), and obese (≥ 20 kg/m^2^), and for 5 years old girls: severely underweight (< 11.8 kg/m^2^), underweight (11.9–12.6 kg/m^2^), normal weight (12.7–16.9 kg/m^2^), overweight (17–18.9 kg/m^2^), and obese (≥ 19 kg/m^2^). The measurement of weight and height were made by the teachers in the school.

With the above presented classification, we classified only 1 premature child in the overweight group and none in obese group, with it being impossible to perform statistical analysis; for this reason, we decided to regroup the sample into 2 groups: severely underweight and underweight in group 1, and normal, overweight, and obese in group 2. With this regrouping, 37.14% of the premature children were in group 1 and 62.86 in group 2. On the other hand, 13.65% of the full-term children were in group 1 and 86.37% in group 2.

Most participants were enrolled in public schools (69.5%) compared with 30.5% of students enrolled in semi-private schools. It should be noted that 65% of children went 1 hour a week to extracurricular physical activities either in the area of gross motor development (such as swimming or dancing) or in the area of fine motor development (such as drawing or painting). In addition, there were no differences in children’s curricula between schools but 35% of them counted on educational support.

### 2.2. Procedure

First, some schools in the Province of Albacete, Spain, were contacted, the objectives of the study were communicated to them, and their participation was requested. Second, the written informed consent were requested to the parents willing to participate, and moreover, the parents answered a questionnaire about the variables of the study including if their children were born premature. Third, teachers at the above-mentioned schools were trained on the usage of the Checklist of Psychomotor Activities (CPA) to apply it properly. Fourthly, teachers of each course made the evaluation of the children with the help of a member of the study team within 1 hour typically used for physical education. Evaluation was based on the observation of the execution of the tasks by the teachers, except the relational affective aspects that were evaluated with their own knowledge of the children. Finally, the data collected from+ the CPA and the parent’s questionnaires were used as the inputs for the study detailed below. The procedure can be seen in Figure 1.

### 2.3. Instruments

To determine the psychomotor development of children, we completed the Checklist of Psychomotor Activities (CPA) [7]. The CPA was applied in a Spanish sample to analyze some psychometric properties such as reliability and validity, but there were no normative scores on the expected developmental parameters for this population [7]. The CPA evaluates the following scales and skills.

The Psychomotor Aspects Scale (PSAS) is composed of 5 factors or skills: laterality (LAT, seven tasks), dynamic coordination (DC, 6 tasks), tonic-postural control (TPC, 3 tasks), motor execution (ME, 3 tasks), and balance (BAL, 5 tasks). LAT is the dominance of one side of the brain in controlling activities or functions; it was measured by tasks such as “hits objects with the left leg”. DC is the ability to flexibly apply skills, knowledge, and situational awareness; it was measured by tasks such as “jump with one foot”. ME is composed of overt and volitional movement, for example, “lies down with his back straight”. BAL is a state of equilibrium or equipoise, measured with actions such as “maintains balance by walking on a curve”.

The Perceptual-Motor Aspects Scale (PEAS) is composed of five factors or dimensions: respiratory control (RC, 3 items), body image and body schema (BI, 4 items), motor dissociation (MD, 3 items), visual–motor coordination (VMC, 6 items), and spatial orientation (SO, 2 items). RC refers to the physiological mechanisms involved in the control of breathing, and was measured by tasks such as “is able to keep the air until it is indicated”. BI is the perception that children have of their physical self and the thoughts and feelings that result from that perception, being measured by tasks such as “distinguishes his own image in the mirror and photographs”. MD is the ability to follow commands from evidence of brain activation in response to motor commands, for example, “is able to use isolated parts of his body when is instructed”. VMC is the coordination of visual perception and fine motor control, for example, “is able to bounce a ball”. Finally, SO is the ability to maintain the body orientation and/or posture in relation to the surrounding environment; it was measured by tasks such as “knows how to situate with respect to an object”.

The Emotional–Social Aspects Scale (ESAS) is composed of 2 factors or dimensions: emotional control (EC, 6 items) and social relationships (SR, 5 items). EC was measured by items such as “expresses himself in an appropriate way adapting to different contexts” and SR for items such as “shows a positive attitude when playing in a group”.

The participants were evaluated by their teachers using the five-level Likert scale where 1 means never and 5 means always. The teachers scored according to the ability of the participants in carrying out the task proposed in each item.

The CPA shows good psychometric properties: reliability (Cronbach’s alpha) with ranges between 0.572 for laterality and 0.872 for balance on the PSAS, between 0.514 (spatial orientation) and 0.825 (respiratory control) on the PEAS, and between 0.572 (emotional control) and 0.800 (social relations) on the ESAS. The reliability of the total scale (which groups all the skills) was very high at 0.935.

### 2.4. Statistical Analysis

The main objective of this study was to compare the psychomotor development and the socio-emotional aspects of the 5-year-old children, taking into consideration their characteristics of being born preterm and full-term. Shapiro–Wilk’s test showed the absence of normality in both groups (probability *p* < 0.000 in all variables), except physical-motor (*p* = 0.027) and affective-relational (*p* = 0.136) in the premature group. For this reason, the differences between groups of preterm and term children were tested with non-parametric test (Mann–Whitney’s *U*).

A secondary objective was to analyze the psychomotor skills both in preterm and full-term children, taking into account their weight measured by current body mass index (BMI). Again, the normality assumption was not met in any group, and thus the analysis of the differences in preterm and full-term children according to BMI was performed using the non-parametric test of Mann–Whitney *U* and median (Me) to fulfill the objectives, we conducted a quantitative, non-experimental, descriptive, cross-sectional study [22].

It was decided that we would use non-parametric statistics because the Shapiro–Wilk normality test indicated the absence of normality for the contrast groups; moreover, the non-parametric procedures of Mann–Whitney *U* and Me allowed us to compare groups of different sizes because they use median and ranges instead of means. A parametric equivalent with fewer contrasts (such as ANOVA) was not used because the assumption of normality was not met.

## 3. Results 

### 3.1. Differences between Preterm and Full-Term According to the BMI

Table 1 and Table 2 present the description of the psychomotor skills in preterm and full-term children, respectively. As can be seen in Table 1 and Table 2, normality assumption was not met. Mean, median (Me), and ranges showed very similar values in premature children when comparing group 1 (severely/moderately underweight) and 2 (normal/overweight/obese); however, some differences were found in full-term children. These differences are precisely analyzed in the next epigraph.

The results of the Mann–Whitney *U* and Me tests indicate significant statistical differences according to BMC only in the full-term children. These differences occurred in the following skills: laterality (median test: *p* = 0.001; *U* test: *p* = 0.000), dynamic coordination (median test: *p* = 0.027; *U* test: *p* = 0.009), sum of physical-motor skills (median test: *p* = 0.011; *U* test: *p* = 0.050), respiratory control (median test: *p* = 0.004; *U* test: *p* = 0.057), visual-motor coordination (median test: *p* = 0.018; *U* test: *p* = 0.001), emotional control (median test: *p* = 0.003; *U* test: *p* = 0.015), and in the sum of affective-relational skills (median test: *p* = 0.017; *U* test: *p* = 0.012). As shown in Table 2, the differences favored the group of normal/overweight/obese children in all skills.

### 3.2. Overall Difference between Preterm and Full-Term Children

Table 3 shows the descriptive statistics related to the overall psychomotor development for children born preterm and full-term. Table 3 shows no significant differences in the variables that describe the physical-motor aspects (laterality, dynamic coordination, motor execution, postural-tonic control, and balance) for premature and non-premature children; however, there were differences in the sum of these values (*U* = 9025.5; *Z* = −2.160; *p* = 0.031) but with a low effect size (*r* = 0.08). On the basis of the median and ranges, we can see that premature children had a lower score in the sum of the physical-motor variables.

Regarding perceptive-motor skills, we found significant differences between premature and full-term children related to body image (*U* = 9025.5; *Z* = −2.388; *p* = 0.017), motor dissociation (*U* = 9120.5; *Z* = −2.210; *p* = 0.027), and visual-motor coordination (*U* = 8810; *Z* = −2.360; *p* = 0.018), although the effect sizes were low (*r* = 0.094, *r* = 0.087, and *r*= 0.092, respectively). There were no differences in respiratory control, spatial orientation, or in the sum of perceptual motor variables. On the basis of the median values and the ranges, we observed that premature children have lower scores on the variables where there were significant differences. Finally, significant differences were found in emotional control (*U* = 8511.5; *Z* = −2.625; *p* = 0.009) and in the sum of social-emotional aspects (*U* = 8880.5; *Z* = −2.289; *p* = 0.022) since full-term children had a higher average range. However, there were no significant differences in social relationships. In this case, the effect sizes were moderate (*r* = 0.103 and *r* = 0.090, respectively).

## 4. Discussion

This study comparatively evaluated children born preterm and full-term, aged five years old, enrolled in the third level of early childhood school. One of the main results of the present research was that prematurity could affect some areas of the psychomotor development such as body image, motor dissociation, visual-motor coordination, and social and emotional aspects. These results are similar to other studies that concluded in the same way—premature children can potentially present a high risk of developing motor problems. These risks refer to those children who, due to pre- or post-natal organic causes, may present a transient interference that could affect their development, introducing prematurity as one of these risk causes [23,24,25].

As shown in the results of the present study, premature children are at a disadvantage, in terms of the sum of physical-motor variables, when compared with children born full-term; this result was also found by other authors [23]. This is because motor development is a set of processes associated with the practice and experiences of the infant, but also with other physical variables such as the premature birth [26,27,28,29].

In relation to the body image and body schema, motor dissociation, and visual-motor co-ordination, the preterm children also presented a lower significant score. The body image and body schema were aspects linked to the perception directly related to the emotional development of children and how they interact with their context [30]. Other studies [30,31,32] also indicated that preterm children have a psychomotor development that is at risk/delayed, with the perceptual aspect being the most affected, leading to schooling problems. Deficiency in motor dissociation and visual-motor coordination are closely related to learning disorders, incompetence in perceptual-motor coordination, clumsiness in manual writing (in the first grades), and difficulties in mathematics [33].

The above-mentioned results allow for professionals to design and evaluate the intervention programs aimed to improve procedures for overcoming specific deficits identified in children born preterm [34,35]. For example, the educators may design a program focused on improving the perceptive skills such as strengthening body image and body self-esteem, practicing visual-motor coordination exercises, or creating activities that promote social interaction of premature children. Regarding motor dissociation, future research may be centered in understanding how the brain of preterm early school-age children dissociates and performs motor tasks.

The preterm children also scored low on emotional control and social development—this lower score is evidenced in the school age, but other authors affirm that it can persist until adulthood [24]. For this reason, it becomes essential to strengthen educational programs in the field of emotional control in order to improve the overall development of children born preterm. Deficiencies in emotional control and in general in all social aspects may be related to their motor immaturity and deficiency in body perception [34]. Another explanation is that premature children suffer various processes at birth depending on their medical condition, the degree of immaturity, and early separation from the mother (for short or very long periods), wherein anxiety states may occur [1].

On the other hand, a second objective of the present research was to determine if the current weight, combined with prematurity, may influence the differences found in psychomotor abilities. Differences only were found in full-term children; those differences indicate that those with severe/moderate malnutrition exhibited less development of some skills such as laterality, visuomotor-coordination, and respiratory and emotional control. It is very interesting that pre-term children with low weight did not present this delay in development that was identified in the full-term children with low weight. In future studies, it would be interesting to delve into the explanation of this result.

The explanation of these results may be the presence of other moderator variables such as education. Early childhood education is important in the detection of special educational needs or alterations in the child’s development. In most of the cases, the teachers are those who detect some of the difficulties in the development of the children by performing a deep assessment in the classroom [28]. In case of observation of alterations in the child’s development of motor skills, these alterations should be communicated to the psychopedagogue, who will refer the child and his/her family to early intervention programs and pediatric centers.

Even though important critical periods of development have already been overcome by the age of five, evaluating children at this stage is essential because it marks the end of a phase and the beginning of the primary educational stage. Prevention at this age is important since the educational rehabilitation of the alterations is more effective due to brain plasticity [29]. Early intervention stimulates the learning processes that will help in the prevention of difficulties and improve the development of children with disabilities or with high risk of having them [30]. The child must be seen from his individuality and attend to his/her needs by accompanying them in their maturity process. The teacher and the pediatrician become observers of this process together with the family, preventing with their practice and actions any difficulties that children may face [31].

It should be noted that the main contribution of this study is the identification of the psychomotor development variables that may be affected in children born preterm when compared to children born full-term at the same age, with the application of the recently developed instrument, the CPA. Using the CPA, most of the variables that have been considered in different theoretical models can be evaluated; in addition, being an instrument designed to be used by teachers, it becomes a powerful tool to establish protocols for preventive purposes in children with high motor risk (specifically focused on the variables of visual-motor coordination, motor dissociation, and strengthening of the body schema).

Another important contribution of this study is the provision of psychometric data obtained from children born preterm and full-term obtained from the comparative study, as well as the age of the selected sample (five years old), since most of the research con-ducted have been with children at very early ages (between zero and three years old), considering the physical-motor aspect and the fact of them being healthy children without severe motor consequences.

The aspects evaluated in this study show how they affect to a lesser or greater degree the aspects related to motor perception and the fundamental emotional aspects present in the acquisition and development of the cognitive processes typical of the primary education stage such as the acquisition of basic core subjects [36] and the development of socio-emotional elements that evidences their immaturity in relation to their motor perception, as well as the detachment that preterm children face when they are separated from their parents while they are in incubators and the consequences evidenced in the present study—all of them vital factors, highlighting the fact that prevention against these conditions or characteristics of being born preterm is key [37].

Despite these important contributions, this study also has some limitations. The results cannot be generalized as the sample is limited to a Spanish geographic region. No specific intervention proposal has been made to improve the psychomotor skills of premature children, but it may be done in future studies. On the other hand, we did not have information on the precise week of birth, only that the minors had been born before week 37; this may have been an important variable to control. Another limitation is the shortage of details on the sample regarding the factors that may moderate the influence of prematurity in the psychomotor development.

The final conclusions of the present work are as follows: (1) Children born preterm were found to have a lower development of some physical-motor skills. (2) In the perceptual-motor field, premature children showed lower scores in the variables related to body image and body schema, motor dissociation, and visual-motor coordination, as well as in socio-relational and affective aspects. (3) The differences between premature and full-term children were not affected by the current weight because the analysis of the BMI indicated no differences in preterm children. (4) It was necessary to establish protocols oriented to the prevention of the difficulties detected in children with psychomotor high-risk and the needs to reinforce the educational programs in this area to improve the integral development of children born preterm.

## Figures and Tables

**Table 1:** Description of variable in premature children (Checklist of Psychomotor Activities (CPA)).

			BMI	Descriptive Statistics				
Scale	Skill	*n*		Mean (SD)	Range	Me	G1	G2
Physical	LT	13	Severely/moderately underweight	24.92 (3.14)	107.35	23	1.692	1.671
		22	Normal/overweight/obese	25.27 (3.60)	118.27	24	1.662	1.893
	DC	13	Severely/moderately underweight	25.38 (4.95)	105.33	27	−1.134	0.284
		22	Normal/overweight/obese	26.18 (3.67)	106.24	27	−0.546	−0.992
	ME	13	Severely/moderately underweight	13.46 (2.33)	134.77	15	−1.412	1.140
		22	Normal/overweight/obese	12.91 (2.63)	132.20	14	−0.008	-0.327
Motor	TCP	13	Severely/moderately underweight	13.69 (2.17)	185.14	15	−1.853	3.067
		22	Normal/overweight/obese	12.32 (2.62)	186.33	12	−0.833	0.128
	BAL	13	Severely/moderately underweight	21.15 (5.44)	143.14	24	−1.320	−0.453
		22	Normal/overweight/obese	20.86 (4.32)	131.29	21.5	−1.342	2.289
	SUM	13	Severely/moderately underweight	98.62 (12.05)	157.88	104	−1.407	0.616
		22	Normal/overweight/obese	97.55 (12.60)	125.21	100	−0.297	−0.428
Perceptive motor	RC	13	Severely/moderately underweight	13.26 (3.19)	117.35	15	1.732	1.262
		22	Normal/overweight/obese	12.83 (2.45)	106.47	13.5	−0.884	−0.552
	BI	13	Severely/moderately underweight	17.62 (5.04)	156.22	20	−2.131	3.365
		22	Normal/overweight/obese	17.41 (3.54)	141.25	19	−1.475	1.164
	MD	13	Severely/moderately underweight	12.62 (3.45)	194.57	15	−1.292	0.154
		22	Normal/overweight/obese	12.14 (3.01)	133.30	14	−0.987	−0.136
	VMC	13	Severely/moderately underweight	23.54 (4.53)	103.04	24	−1.380	1.663
		22	Normal/overweight/obese	24.73 (6.08)	131.09	26.5	−1.566	−1.193
	SO	13	Severely/moderately underweight	9.00 (1.29)	133.04	10	−1.410	−1.824
		22	Normal/overweight/obese	8.91 (1.99)	131.39	9.5	−0.913	−1.149
	SUM	13	Severely/moderately underweight	76.00 (15.27)	128.98	82	−1.884	2.514
		22	Normal/overweight/obese	76.05 (14.37)	125.27	82	−0.919	−0.478
Emotional–social	EC	13	Severely/moderately underweight	24.85 (4.53)	133.14	26	−1.310	0.616
		22	Normal/overweight/obese	23.41 (6.08)	121.49	24	−1.066	0.665
	SR	13	Severely/moderately underweight	19.46 (3.30)	133.04	21	−1.650	1.864
		22	Normal/overweight/obese	18.82 (4.22)	131.39	18.5	−0.319	0.498
	SUM	13	Severely/moderately underweight	44.31 (7.13)	133.98	47	−1.704	2.294
		22	Normal/overweight/obese	42.23 (10.36)	115.27	42	−0.719	0.068

Note: LT = laterality; DC = dynamic coordination; ME = motor execution; TPC = tonic-postural control; BAL = balance; RC = respiratory control; BI = body image; MD = motor dissociation; VMC = visual motor coordination; SO = spatial orientation; EC = emotional control; SR = social relationships.

**Table 2:** Description of variables in full-term children (Checklist of Psychomotor Activities (CPA)).

			BMI	Descriptive Statistics				
Scale	Skill	n		Mean (DT)	Range	Me	G1	G2
Physical-motor	LT	87	Severely/moderately underweight	25.16 (4.77)	106.25	23	0.533	0.036
		550	Normal/overweight/obese	27.49 (5.12)	174.84	27	−0.014	−1.002
	DC	87	Severely/moderately underweight	26.25 (3.57)	147.45	27	−0.897	0.329
		550	Normal/overweight/obese	27.31 (3.11)	121.32	29	−0.195	−0.647
	ME	87	Severely/moderately underweight	13.82 (1.92)	174.17	15	−1.982	3.866
		550	Normal/overweight/obese	13.84 (2.24)	123.20	14	−1.607	−3.196
	TCP	87	Severely/moderately underweight	13.21 (2.58)	185.24	14	−1.053	0.391
		550	Normal/overweight/obese	12.90 (2.03)	179.13	13	−0.266	−0.180
	BAL	87	Severely/moderately underweight	21.82 (3.86)	184.34	23	−1.410	0.183
		550	Normal/overweight/obese	21.46 (3.36)	141.19	22	−0.666	−0.080
	SUM	87	Severely/moderately underweight	100.25 (11.85)	148.98	104	−1.347	2.644
		550	Normal/overweight/obese	103 (10.71)	114.37	102	−0.883	1.809
Perceptual-motor	RC	87	Severely/moderately underweight	13.34 (2.46)	107.25	15	−1.477	1.572
		550	Normal/overweight/obese	13.09 (2.24)	106.37	14	−1.144	0.762
	BI	87	Severely/moderately underweight	18.62 (2.87)	147.12	20	−2.301	4.745
		550	Normal/overweight/obese	19.13 (2.00)	127.55	20	−1.745	8.047
	MD	87	Severely/moderately underweight	13.49 (2.36)	134.57	15	−1.869	1.725
		550	Normal/overweight/obese	13.65 (1.68)	133.80	14	−0.787	0.763
	VMC	87	Severely/moderately underweight	24.95 (4.28)	185.04	25	−0.701	−0.067
		550	Normal/overweight/obese	26.72 (3.30)	188.13	27	−1.295	0.542
	SO	87	Severely/moderately underweight	8.85 (3.73)	133.04	9	−2.130	4.983
		550	Normal/overweight/obese	9.36 (4.11)	148.09	10	−2.273	2.419
	SUM	87	Severely/moderately underweight	79.26 (9.70)	128.98	82	−1.764	2.781
		550	Normal/overweight/obese	81.95 (8.57)	134.27	84	−1.339	6.148
Emotional–social	EC	87	Severely/moderately underweight	25.74 (3.63)	137.22	26	−1.352	0.258
		550	Normal/overweight/obese	26.86 (3.66)	140.53	28	−1.269	4.352
	SR	87	Severely/moderately underweight	20.21 (2.89)	134.25	21	−0.568	0.183
		550	Normal/overweight/obese	20.76 (3.08)	133.95	21	−0.289	1.259
	SUM	87	Severely/moderately underweight	45.94 (5.87)	138.12	47	−1.224	2.487
		550	Normal/overweight/obese	47.29 (6.15)	141.04	49	−0.690	3.348

Note: LT = laterality; DC = dynamic coordination; ME = motor execution; TPC = tonic-postural control; BAL = balance; RC = respiratory control; BI = body image; MD = motor dissociation; VMC = visual motor coordination; SO = spatial orientation; EC = emotional control; SR = social relationships.

**Table 3:** Descriptive statistics for children born preterm and full-term.

			Descriptive Statistics				
Scale	Skill	Prematurity Condition	Mean (SD)	Range	Me	G1	G2
Physical-motor	LT	Full-term	25.14 (3.39)	24	288.40	1.632	1.620
		Preterm	25.14 (3.39)	24	288.40	1.632	1.620
	DC	Full-term	27.14 (3.20)	28	350.08	−1.189	1.045
		Preterm	25.89 (4.14)	27	289.04	−0.927	0.100
	ME	Full-term	13.81 (1.56)	14	348.68	−1.669	−1.100
		Preterm	13.11 (2.50)	15	315.43	3.423	−0.075
	TCP	Full-term	12.95 (2.12)	13	346.93	−1.010	0.928
		Preterm	12.83 (2.52)	14	348.29	−1.061	0.383
	BAL	Full-term	21.49 (3.44)	22	347.41	−0.734	0.023
		Preterm	20.97 (4.69)	22	339.20	−1.259	0.995
	SUM	Full-term	102.35 (10.69)	103	350.78	−0.943	1.867
		Preterm	97.94 (12.23)	101	275.87	−0.627	−0.322
Perceptual-motor	RC	Full-term	13.14 (2.29)	14	346.50	−0.627	−0.322
		Preterm	13.00 (2.71)	15	356.49	−1.239	0.399
	BI	Full-term	19.04 (2.13)	20	350.46	−1.706	−1.805
		Preterm	17.49 (4.09)	20	281.87	14.052	2.320
	MD	Full-term	13.63 (1.79)	14	350.64	−1.732	4.075
		Preterm	12.31 (3.14)	14	278.59	−0.918	−0.628
	VMC	Full-term	26.44 (3.52)	27	351.11	−0.908	−2.902
		Preterm	24.29 (5.51)	26	269.71	−1.268	1.218
	SO	Full-term	9.29 (1.38)	10	349.45	−1.110	0.453
		Preterm	8.94 (1.34)	10	300.99	0.398	0.778
	SUM	Full-term	81.53 (8.86)	83	350.39	−1.488	5.618
		Preterm	76.03 (14.49)	81	283.29	0.999	0.198
Emotional–social	EC	Full-term	26.36 (3.60)	27	351.56	3.104	23.94
		Preterm	23.94 (5.74)	25	261.19	−1.244	1.360
	SR	Full-term	20.61 (2.83)	21	349.19	−1.244	1.360
		Preterm	19.80 (3.49)	21	305.87	0.966	0.800
	SUM	Full-term	46.97 (5.83)	48	351.00	−1.244	5.253
		Preterm	40.75 (8.70)	45	271.73	1.360	0.611

Note: LT = laterality; DC = dynamic coordination; ME = motor execution; TPC = tonic-postural control; BAL = balance; RC = respiratory control; BI = body image; MD = motor dissociation; VMC = visual motor coordination; SO = spatial orientation; EC = emotional control; SR = social relationships.

## Data Availability

The data presented in this study are available on request from the corresponding author.
